# Managing Matajoosh: determinants of first Nations’ cancer care decisions

**DOI:** 10.1186/s12913-016-1665-2

**Published:** 2016-08-18

**Authors:** Josée G. Lavoie, Joseph Kaufert, Annette J. Browne, John D. O’Neil

**Affiliations:** 1 MFN – Centre for Aboriginal Health Research, University of Manitoba, #715, 727 McDermot Avenue, Winnipeg, MB R3P 3E4 Canada; 2 Department of Community Health Sciences, University of Manitoba, College of Medicine - University of Manitoba, Room S113 - 750 Bannatyne Avenue, Winnipeg, MB R3E 0W3 Canada; 3 UBC School of Nursing, T201 2211 Wesbrook Mall, Vancouver, BC V6T 2B5 Canada; 4 Faculty of Health Sciences, Simon Fraser University, Blusson Hall, Room 11300, 8888 University Drive, Burnaby, V5A 1S6 BC Canada

**Keywords:** Indigenous, Canada, Health equity, Access, Primary care, Primary healthcare

## Abstract

**Background:**

Accessing cancer treatment requires First Nation peoples living in rural and remote communities to either commute to care, or to relocate to an urban centre for the length or part of the treatment. While Canadians living in rural and remote communities must often make difficult decisions following a cancer diagnosis, such decisions are further complicated by the unique policy and socio-historical contexts affecting many First Nation peoples in Canada. These contexts often intersect with negative healthcare experiences which can be related to jurisdictional confusion encountered when seeking care. Given the rising incidence of cancer within First Nation populations, there is a growing potential for negative health outcomes.

**Methods:**

The analysis presented in this paper focuses on the experience of First Nation peoples’ access to cancer care in the province of Manitoba. We analyzed policy documents and government websites; interviewed individuals who have experienced relocation (*N* = 5), family members (*N* = 8), healthcare providers and administrators (*N* = 15).

**Results:**

Although the healthcare providers (social workers, physicians, nurses, patient navigators, and administrators) we interviewed wanted to assist patients and their families, the focus of care remained informed by patients’ clinical reality, without recognition of the context which impacts and constrains access to cancer care services. Contrasting and converging narratives identify barriers to early diagnosis, poor coordination of care across jurisdictions and logistic complexities that result in fatigue and undermine adherence. Providers and decision-makers who were aware of this broader context were not empowered to address system’s limitations.

**Conclusions:**

We argue that a whole system’s approach is required in order to address these limitations.

## Background

Matajoosh is an Ojibway word for ‘cancer’, which translates as “worm eating at your insides” [1, p. 457]. Hart-Wasekeesikaw [2] and Orchard [3] explored the Anishinaabe metaphor of “*manitoch*” (cancer as worm), *manitosis* (worm, spider-like bug) and *manicosak* (maggots) that are used in some communities to explain the devastating effects of the disease. Since 1991, cancer has become the leading cause of death among Canadian First Nation males and the second leading cause among First Nation females. In the province of Manitoba, where data on relocation for First Nation peoples for cancer care was collected, increases in cancer incidence have been more modest (7 %) but increases in premature mortality from cancer remains a concern [4]. A recent study by Torabi and colleagues [5] reported that although colorectal cancer (CRC) related mortality is on the decline in Manitoba, this decline is only experienced by individuals of higher socio-economic status. Manitoba residents of lower socio-economic status, which includes most First Nations, are in fact showing an increase in mortality from CRC [5]. Studies have repeatedly reported that First Nation peoples have poorer survival rates once diagnosed with cancer [6, 7]. Although scholars have documented the experience of Australian Aboriginal peoples in the pursuit of cancer care [8–10, for examples], similar work has yet to occur in Canada.

Accessing cancer treatment requires First Nation individuals living in rural and remote communities to either commute to care, or to relocate to an urban centre, in this case the capital, Winnipeg, for the length or part of the treatment. An alternative is for the patient to decide not to pursue acute cancer care. While Canadians living in rural and remote communities must often make difficult decisions following a cancer diagnosis, such decisions are further complicated by the unique policy and socio-historical contexts affecting many First Nation peoples in Canada. These contexts often intersect with negative healthcare experiences [11, 12] and jurisdictional confusion encountered when seeking care [13]. Given the rising incidence of cancer within First Nation populations, there is a growing potential for negative health outcomes.

In our initial overview publication, we document First Nation peoples’ experiences of medical relocation, highlighting policy-related issues [13]. Ambiguity and discontent in policy and mid-level decision-making have been longstanding problems for First Nation peoples living in rural and remote regions of Canada [14], made recently more complex because of shifts in accountability that resulted in more sharply defined and less flexible policies, undermining decision-makers’ discretion, pragmatic compromises and responsiveness [13]. In this paper, we take a closer look at the particular set of contexts that influence First Nation peoples’ access to and experience of cancer care. This is our entry point for analysis in this paper. Our objectives are two fold. First, we critically explore how the contexts of peoples’ lives intersect with structural barriers to shape peoples’ access to care and expectations of cancer care. Second, we highlight policy issues shaping healthcare experiences and outcomes. We propose to foreground the voices of patients and family members in explaining their own or their family members’ cancer care journey, and complement these perspectives with those of providers.

The next section provides an overview of the methods used to gather and analyse the evidence discussed in this paper. The research findings are described in two sections that summarize the context of cancer care in Manitoba, based on the cancer journey of participants and the perspective of providers. The last section discusses key themes, implications for policy and healthcare delivery, and conclusions.

### The jurisdictional context of cancer care in Manitoba

Manitobans access cancer care from a spectrum of relatively autonomous and at times, loosely integrated networks of service providers, including Family Physicians (FPs) and specialists working in publicly funded private practice. In addition, CancerCare Manitoba (CCMB) is responsible for providing care, treatment and support across the entire cancer service spectrum. If living on-reserve, the first point of care is generally the on-reserve clinic [see 15 for a detailed description], which is federally funded by the First Nations and Inuit Health Branch of Health Canada (FNIHB, the federal authority that funds health services on-reserve and approves access to medical transportation) and in most cases, locally managed by a First Nation authority. In Manitoba 15 % of the population identifies as First Nations. Services at on-reserve Nursing Stations and Health Centres are generally provided by part time or full time nursing staff, community health services (primary prevention, immunization), supported by locally hired paraprofessional providers who assist with planning clinics, home care, education and cultural liaising. Some larger communities have full time nursing staff, who in addition to the above, also provide some primary care services, supplemented with visiting primary care and specialist clinics. The federal-provincial jurisdictional divide is embedded in the Canadian Constitution, and likely to remain for years to come. Jurisdictional ambiguities have been repeatedly documented across Canada [16] and in Manitoba [17], leading to discontinuities of care and logistical fatigue when coordinating and seeking care [13].

Although participation in cancer screening programs has improved over time, especially for cervical cancer screening [18], barriers to early detection remain. For First Nations living on-reserve, screening programs (with the exception of cervical cancer screening), which are currently understood as a matter of provincial jurisdiction, are not accessible on-reserve. Although the federally funded Non-Insured Health Benefit (NIHB) program assists First Nations living on-reserve with medical transportation expenses when care is required, this program does not support transportation for screening purposes [6]. This program instead is limited to transportation for emergency care, and medically necessary care, as determined by a FP.

Some communities receive primary care services from provincially funded visiting FPs (often “fly-in” in remote communities). Anecdotal evidence however suggests that needs often exceed supply, and that some visiting FPs provide “walk-in clinic” type of care during a visit expecting First Nations to access “their regular” FP for problems requiring further investigation, not realizing that most First Nations do not have a regular FP ensuring continuity of care. Challenges related to the recruitment and retention of FPs willing to work in rural and remote communities undermine rural residents’ access to first contact care in a timely manner, and results in poorer quality and continuity of care [19]. Jurisdictional fragmentation, the poor integration of existing services, and high staff turnover where this is an issue, add additional complexities [20].

There is some documented evidence that First Nations diagnosed with breast cancer are often diagnosed at a later stage of the disease [21]. A lack of data on ethnocultural identity in cancer datasets prevents a more comprehensive understanding of the situation. The barriers to accessing screening discussed above are a likely factor. Once diagnosed, treatment may require a combination of surgeries, cancer drugs, chemotherapy, and/or radiation therapy. In Manitoba, where this study is located, some treatment may be accessed on an out-patient basis, in regional centres (off-reserve), but the majority of treatment modalities are accessible only in tertiary care facilities located in Winnipeg. These services are under the purview of the provincial government and regional health authorities. An exception is out-patient access to some prescription medication, which is provided by the federally funded NIHB program. Palliative care is a possible end point in the continuum of care, once treatment options are exhausted. In Manitoba, palliative care and especially pain control, is available in urban and to a limited extent in regional centres [22], but remains unavailable on-reserve. A study of 692 palliative care telephone consultations in British Columbia documented that nearly one third of all calls came from patient living in communities with a population under 5000. Almost half (49 %) of these consultations were for pain management [23], suggesting unmet needs in rural and remote communities. This context shapes First Nation peoples’ experience of cancer care, and provides a necessary backdrop to this study.

## Methodology

This paper reports selected findings from a larger study that aimed to a) document the policy framework that currently shapes the experience of medical relocation/multilocality; and b) document the experience of First Nations as they negotiate jurisdictional boundaries and navigate the health care system. For this study, we partnered with the *Nanaandawe Wigamiq* – First Nation Health and Social Secretariat of Manitoba (a First Nations political organization formed in 1988 to advocate on issues that affect all First Nations in Manitoba) and four First Nation communities. Ethical approval was received from the Health Research Ethics Board of the University of Manitoba (HS11445-H2009:189). In each community, a partnership agreement was signed by the research team and the community leadership, detailing the purpose and process of the study.

The overall study was informed by interpretive inquiry, which is an understanding of knowledge as socially constructed [24]. A total of 129 people participated in the study. Patients and family members comprised the largest group at 70 participants. The perspectives of providers and decision-makers were documented in 59 interviews. As interviews unfolded, we continuously monitored the dataset for breath, to adequately reflect the complexity and nuances reflected in the participants’ experiences across different dimensions, including policy, healthcare trajectories, rural and urban contexts, and across different conditions health conditions.

Participating patients and family members reported that they had experienced relocation, either permanently or temporarily, to access specialized services including dialysis, cancer, specialized maternal and child care, cardiovascular care, rheumatoid arthritis and because of a new or existing disability. The most common type of relocation mentioned was related to renal failure and the need to access dialysis: this is the object of a forthcoming paper. Findings from the broader dataset have already been published [13], and provide a necessary context for this analysis.

This paper draws on interviews conducted with patients, family members and healthcare providers whose relocation experiences were directly related to seeking cancer care. This included in-depth, open-ended interviews with 16 patients or family members (12 women and 4 men) who spoke about their own healthcare experiences or those of family members with whom they were closely involved. We also completed 15 in-depth interviews with rural and urban health service providers. Characteristics are shown in Tables 1 and 2.

**Table 1:** Patient interviews, characteristics

Interview number	Age	Gender	Relating the story of	Type of cancer	Other morbidities discussed	Trajectory of care	Comm & type^b^	Outcome
002	59	Male	Self	Leukemia	Heart condition, diabetes	Traveling back and forth to care^c^	C, NI/SI	No active disease reported^a^
005	50	Male	Female partner	Breast	None mentioned	Traveling back and forth to care^c^	C, NI/SI	Died in a regional hospital
			Male child	Leukemia	None mentioned	Moved to Winnipeg	C, NI/SI	Active treatment
007	47	Female	Self	Uterus, stomach	Diabetes renal failure	Traveling back and forth to care^c^	C, NI/SI	Active treatment
028	55	Female	Male partner	Lung cancer	Diabetes renal failure	Traveling back and forth to care^c^	D, I	Died at home, in the city
029	60	Female	Daughter	Liver	None mentioned	Moved to Winnipeg	D, I	Died in temporary accommodations, the city
031	N/A	Female	Sister	Not specified	None mentioned	Moved to Winnipeg	D, I	Died at home, in home community
			Self	Colon	None mentioned	Traveling back and forth to care^c^	D, I	No active disease reported^a^
033	N/A	Male	Sister	Not specified	None mentioned	Traveling back and forth to care^c^	D, I	Died in hospital, in the city
034	60	Male	Female partner	Stomach	None mentioned	Traveling back and forth to care^c^	D, I	Died in hospital, in the city
050	39	Female	Self	Uterus	None mentioned	Traveling back and forth to care^c^	A, NI/SI	No active disease reported^a^
208	66	Female	Daughter	Breast	None mentioned	Traveling back and forth to care^c^	D, I	Active treatment
209	36	Female	Grand-mother	Colon	Frailty	Moved to Winnipeg	D, I	Died in assisted living facility, in the city
			Father	Multiple myeloma	Diabetes	Decided not to pursue cancer care	D, I	Died at home, in home community
210	45	Female	Mother	Not specified	None mentioned	Moved to Winnipeg	D, I	Died at home, in home community
213	45	Female	Self	Breast	None mentioned	Traveling back and forth to care^c^	D, I	No active disease reported^a^

^a^As reported at the time of the interview

^b^NI/SI: Non-isolated or semi-isolated community. I:Isolated community, Letters refers to community characteristics outlined in Table 3

^c^Once released from the hospital. In two cases, hospitalization in the city was long term (6 months), but once discharged, they came home and commuted to care

**Table 2:** Provider characteristics

Interview number	Gender	Category	Title
001	Female	On-reserve	Nurse supervisor
018	Female	Urban	Patient navigator
020	Female	Urban	Discharge coordinator
023	Male	Urban	Translator
025	Female	Urban	Social worker
036	Female	Urban	Health services coordinator
038	Female	On-reserve	Health director
039	Female	On-reserve	Transportation clerk
040	Male	Urban	Care coordinator
052	Female	On-reserve	Home care nurse
055	Female	Urban	Palliative care coordinator
056	Male	Urban	Director, Family physician services
205	Female	On-reserve	Home care worker
214	Female	On-reserve	Nurse
502	Female	On-reserve	Home care worker

Interviews were conducted in Winnipeg with First Nation peoples from any of the Manitoba 63 First Nation communities, and in 4 First Nation communities, following a purposive sampling framework. This approach is well suited to the exploratory design used in this study, and was an effective method for ensuring the inclusion of participants who could discuss a diverse range of experiences. Providers were identified using a respondent-driven sampling process. To start, a group of key providers were identified by the research team and stakeholders based on their role and expertise in the healthcare system (for example, Patient Navigator) and were asked to suggest other providers who could be approached to provide narrative data on relocation. In Winnipeg, patients were contacted through the Patient Navigator program of the Nanaandawewigamig First Nation Health and Social Secretariat. In communities, interviews with patients and family members were conducted at the community Health Centre, which is often a focal point in the community. Healthcare providers were asked to help identify individuals fitting our criteria of having experience accessing health services outside of the community for a significant event (cancer, dialysis, rehabilitation following a stroke or car accident, etc.) We specifically asked to interview parents of children who required care for significant periods of time. A consent form was provided and explained to the participant. We repeatedly reassured participants that their participation was entirely voluntary and that they could withdraw from the study at any time. Many patients and family members stated that they were eager to tell their story. Pseudonyms have been given to communities to protect privacy. Characteristics are shown in Table 3.

**Table 3:** Participating First Nation community characteristics

	Local services	Population on-reserve (2010)	Level of care accessible locally	Closest point of care	Distance to Winnipeg
Community A	Health Office	Between 750–1000	Part-time workforce, screening and prevention services only.	Between 50 and 100 km, local hospitals, FPs	Over 600 km
Community B	Health Office	Between 1250–1500	Part-time workforce, screening and prevention services only	Between 50 and 100 km, local hospitals, FPs	Between 250 and 300 km
Community C	Health Centre	Between 1250–1500	Emergency, screening and prevention available 5 days per week, with limited or no after hour care locally.	Under 10 km, local hospital, FPs	Over 600 km
Community D	Nursing station	Between 1000–1250	Primary healthcare treatment and prevention, accessible 24/7.	Winnipeg (no road access)	1 h flight

All interviews were conducted by members of the research team (JL, JK, AB) and/or by a senior Research Associate. Interviews lasted on average 45 min. All interviews were digitally recorded, transcribed verbatim, cleaned of any personal identifiers, and compared with the audio recordings for technical accuracy. Using interpretive thematic analysis for qualitatively-derived data, the team reviewed the transcripts to identify concepts, processes, and linkages to theoretical perspectives as well as any recurring and contradictory patterns in the data. NVivo 10, a qualitative data analysis software, was used by two Research Assistants to independently code and organize the interview data, using the code book (one for patients and one for providers) developed by the research team. The code books were periodically reviewed and discussed by the research team, and compared to independent coding of transcripts completed by research team members, to ensure consistency in the coding process. Over time, analysis shifted to a more abstract and conceptual representation of the processes and themes reflected in the data. Five broad themes are reflected in the data, related to, reasons for and trajectory of relocation (for examples, type of care needed, multi-relocations), care experience (for example, continuity, discontinuity, responsiveness); basic needs (housing, transportation, food); shifting social role (loss of social and cultural status, isolation, family dynamics).

Credibility of the analysis was continually evaluated by members of our research team, who included experts in ethnographic research, PHC services, First Nation health and health equity. Preliminary results were presented to First Nation Health Technicians Network (a group of First Nations Health Directors to advise on interpretation). In these meetings, on-reserve healthcare providers affirmed that the themes reflected in the data resonated with their experience working with families and patients in the healthcare sector. Considerable theme overlap was found between interviews conducted with patients and family members. These are presented together.

## Results

From the time of diagnosis, patients who decided to seek or undertake cancer care (all except one in this dataset) travelled to Winnipeg to access care. Their length of stay varied. In two cases, care required a lengthy hospitalization (Patients 002, 029). In most others, hospitalization was brief and often involved surgery. Outpatient chemotherapy or radiation treatments followed. Only one person mentioned that chemotherapy was accessible in a regional centre closer to their own home community (Patient 005). For all others, initial care was accessed in Winnipeg.

Some were successfully treated and recovered (Patients 02, 31, 50, 213). At the time of the interview, some suspected or knew that their cancer had recurred. Family members also related the story of loved ones who had died (Patients 05, 28, 29, 31, 33, 34, 208, 209, and 210). Many of the patients and family members’ stories of cancer journeys followed a similar pattern. Key factors in patients’ decision-making are summarized in Table 4 below.

**Table 4:** A patient’s journey, in the context of late diagnosis/referral

Care trajectory	Basis of treatment decision	Challenges	Maintaining connection with home and family
Refused care	• Wanting to die at home	• Formal and informal home care • Pain control • Forced hospitalization at end stage	• Less of an issue until end stage
Traveling for care	• Connection to family • Maintaining employment	• Access to medical transportation and financial support for appointments • Living in hotels while on chemo	• Less of an issue until end stage if terminal
Relocating	• Safety • Better access	• Access to medical transportation and financial support for appointments • Access to adequate accommodations	• Isolation from family • Barriers to coming home

### Challenges associated with patients navigating the system

Front line workers play a critical role in the care experience of patients. All the healthcare providers (social workers, physicians, nurses, patient navigators, and administrators) we interviewed in this study were committed and compassionate individuals who wanted to assist patients and their families. One of the most prevalent issues raised in the provider interviews was the need for patients to have access to more fully integrated services, and the importance for patients of having strong family support to mitigate challenges associated with access care and the impact of “logistical fatigue” in pursuing care. One senior provider commented:

[M]y colleagues were blown away about where to even start to resolve [returning home], both from a policy point of view, a human resources point of view, a facility resource point of view… [G]oing from a northern nursing station by dedicated air ambulance to a tertiary care centre: we seem to have that just about right. But there seemed to be huge difficulties in policy and procedures in sending somebody home to receive care (Provider 056).

Policy challenges are related to the fragmented context of care that requires First Nations to “cross” jurisdictional boundaries repeatedly to access care. This crossing back and forth is rarely seamless, generally requires advocacy by healthcare providers and often ends in delays while awaiting approvals.

In Winnipeg, the Patient Advocate Unit at the Assembly of Manitoba Chiefs, and patient advocates and social workers from the Winnipeg Regional Health Authority (WRHA), advocate for patients to access necessary services such as housing, food, transportation, insurance coverage for medication and patient discharge plans (Provider 18). Patient navigator programs have been shown elsewhere to have a significant positive impact on continuity of care [25]. However, the social workers and patient navigators we interviewed indicated that their case load was overwhelming (Providers 18, 20).

Some providers such as discharge planners discussed their role in assessing the patient’s needs, and working with the patient’s community to ensure continuity after discharge. These providers told us that despite jurisdictional fragmentation, it was still possible for service providers to work together as a team to safely relocate patients back to their communities (Providers 1, 20).

[F]or palliative – like cancer… we try and accommodate again as much as we possibly can. Now, [FNIHB] has a really – in the last couple cases here in the last few months that they’ve participated in, trying to accommodate the population to go back to the First Nations communities… And no matter what, whether you’re – whether it’s jurisdiction, it’s not so much argument that we – you know, it’s not an argument of who pays what; it’s trying to work together to have the patients safe and to provide what that is they need (Provider 020).

For some First Nation peoples living in rural and remote communities, the prospect of cancer care can get overshadowed by logistical challenges associated with relocating, navigating the multiple complex but necessary activities of city living (transportation, safe housing, affordable food for examples) and managing confusing and at times inconsistent jurisdictional complexities. Providers described how family members often played an essential role in helping to prepare family members for urban experiences, support them through their care, liaise with the extended family and advocate for them:

And then, they might have one family member that will go and stay with them. And oftentimes, they’re taking turns….And most of the time, the families are the ones that will look at their situations and say, “You know what? I think this – my niece over here: she’s not working. She doesn’t have any children. She’s not – she doesn’t have to worry about other kids or dependents. So she’s probably the best one to come with me to Winnipeg and stay with me.” So then, this young girl goes and stays with that parent and stays with them while they’re getting their – and then communicates over here with family – what’s going on. And if the person gets into a stage where they’re very ill or need some support, she’ll be phoning and saying, “You know, I need you to come here because our Mom is not doing well. I need some support” (Provider 001).

Taking turns can result in complexities for family members, who may have to act as advocates while learning how to navigate a multijurisdictional healthcare system fraught with complexities and contradictions.

### Challenges to obtaining timely diagnoses

As indicated in Table 1, all of those who relocated, with the exception of a child with leukemia (Patient 005), reported advanced disease state at the time of diagnosis, and subsequently died. While some late diagnosis may be related to fear and avoidance, those interviewed for our study related multiple attempts at seeking care locally to address a health concern (most often recurring pain), until an acute episode led to an emergency transfer to an urban centre, and a cancer diagnosis.

She used to get sick, she used to come [to the on-reserve health clinic], but they didn’t send her out right away. They tried to treat her – like, gall bladder attack or something like that - bladder infection, kidney infection. So she wasn’t properly diagnosed here, till she really got sick. And then they Medicaved [emergency transfer] her out. And then she was in the hospital [for 3 months before dying] (Patient 029).

But she came [to the on-reserve nursing station] so many times and they would just say, “Well, that’s just a cyst,” till it became a cancer… (Patient 208).

Delays in diagnosis are linked to two policy-related factors: the limited level of care accessible in First Nation communities, where a cancer diagnosis is beyond the scope of services provided; and a federal medical transportation policy that will not subsidize transportation for preventive and diagnosis purposes.

Some patients may opt to not pursue active cancer care treatment. In some cases, patients may also refuse to pursue palliative care off-reserve, when told that their cancer is too advanced to benefit from acute treatment. Refusal may however occur when acute treatment might still be an option. One family member talked about her father, who refused to relocate, and lived at home with cancer for 5 years (Patient 209). Two rural providers (Providers 052, 502) explained that patients’ rationale for not pursuing care was often linked to having to leave their community for extended periods of time. Providers suggested that patients were afraid to die away from home (as was the case for Patient 209), or to be unable to choose to come home should they want to, because of the shortage of housing on-reserve or a lack of financial resource to afford coming home.

Our findings suggest that delayed diagnoses related to the level of care accessible on-reserve and a policy that does not support medical transportation costs associated with preventive care result in poorer outcomes, perpetuating patients’ view that a cancer diagnosis is invariably terminal. A fear of a terminal diagnosis may result in delays in seeking care. Federal and provincial disputes over their responsibility for coverage of cancer care drugs for First Nations^1^ add unnecessary logistical complexities, stress and may promote non-adherence or a refusal to seek care.

### The lack of attention to peoples’ material circumstances, logistical burden and fatigue

Our interviews suggest that those who can travel back and forth for care were those initially diagnosed with less advanced disease. Traveling back and forth was the solution preferred by patients, when possible. Considerable hardship was nevertheless reported, mostly linked with needing to arrange medical transportation (Patient 050, Providers 025, 039), traveling alone long distance while sick, finding suitable accommodations while receiving treatment in the city (Patients 028, 059), and finding support in the city.

One patient reported persistent medical transportation-related challenges associated with attending her appointments:

[I]t was coming to the point where I was cancelling my appointments. Because… like, there was no way getting there. And sometimes they’d [the federal clerk tasked to arrange medical transportation for First Nation patients] go, “Oh, we don’t have the money. We can’t send you.”. It was, like, more or less choices where I had to send myself and hire people with my own monies kinda thing, just to go to my appointments (Patient 050).

When transportation was available, it often involved hardship for vulnerable patients and family members:

Well, we had another patient that used the [medical transportation] van. But he wasn’t really too comfortable riding in there because of his medical condition…. Sometimes he would have to get off. One time, he got off in [the next community] – that convenience store there, ‘cuz he couldn’t handle the ride (Provider 039).

[W]hy are patients driving themselves in with stomach cancer, eating Tylenol because they feel like crap?” (Provider 025).

People also faced significant challenges related to housing, while receiving outpatient treatment. This was particularly problematic for people without family members living in Winnipeg, who might otherwise offer support with housing or transport. FNIHB provides some support to house patients that require care away from their community, for a period of 4 months [26]. FNIHB issues yearly tenders, and contracts directly with local hotels and hostels. Selected bids are related to limited federal budgets, resulting in “economical” options:

And they pick the crummiest places that I wouldn’t – I couldn’t – you now, something you’re not used to. And for me personally, if I had a choice, I wouldn’t pick the hotels that they have … and there’s a lot of panhandlers there and it’s – it wasn’t as clean as I wanted, for somebody that was terminal (Patient 028).

In contrast, patients who did have family members living in Winnipeg were better able to make arrangements tailored to their own need.

She just finished her chemo treatments last week, I think it was. And she – like, it was for a 5-week period. She… was staying in Winnipeg during that time. But then, she – she would get a ride back, like, on a Monday to go and do treatment during the week. Then she would come home on weekends and then go back on Monday. She stayed with family (Provider 059).

Although eligibility is at times contested because of missing birth certificates, or due to jurisdictional confusion (Lavoie et al., 2014), First Nations relocating to an off-reserve centre for care can access Income Assistance from provincial authorities. In one case, while her daughter was sick and on chemotherapy, a mother called FNIHB and was told “We cannot provide you transportation anymore because [your daughter is] living in the City” (Patient 029). So the mother had to buy a car. The same person reported what happened to her daughter when she attempted to seek Income Assistance: “And then we had to drive her – for help at the Welfare office and she was suffering [with advanced cancer], sitting there… About 3 h we waited there in line” (Patient 029). As one provider explained:

They always have the red tape; it’s what it is. We have to contact this person, then this person, then this person, then this person. And sometimes the information is lost in the shuffle. Then you have to start all over again (Provider 205).

The above quotes show that despite a safety net (Income Assistance, medical transportation support, etc.) in place to support First Nations accessing care in the city, accessing these resources is difficult, and there are considerable limitations to what is provided. Patients and family members emphasized having to be assertive to receive appropriate care and associated supports. Being assertive can however be difficult given past and often intergenerational experiences of dismissal when seeing healthcare services [11, 13]. Some of the patients interviewed in this study described these challenges:

[L]ike, with [FNIHB]: I think… they don’t really care… They give you the run-around (Patient 210).

She had – she has cancer in the breast. And she just found out now she’s gonna do radiation. She’ll be in Winnipeg for a month… But she’s – but I taught her, like, “Speak up.” You know? “Speak up. Don’t be afraid to – to ask for something, even if you offend somebody. Just tell them, you know – just tell them just what you want.” You know? (Patient 208).

### Balancing wanting to be home with the need for care

One of the most prevalent issues described by patients and their families reflected peoples’ fears of dying away from home. In all cases, challenges associated with balancing treatment requirements with family and cultural obligations were mentioned. Being home was connected to fulfilling cultural obligations (in ceremonies, as a member of an extended family), and being able to maintain personal and cultural safety (not having to encounter discrimination in everyday activities, not having to worry about the impact of interpersonal or structural violence in the city, having family and friends to help). In some cases, the importance of retaining family and community connections resulted in significant compromises over care decisions. This was particularly evident in the context of follow-up after remission, as this patient described.

Like, if it were up to me, if I didn’t have grandchildren - like, I wouldn’t wanna take my grandchildren to Winnipeg; now it’s so scary. Like, but if it were up to me, I’d go move back to Winnipeg and I’d stay there. And, like, I’d live there, just knowing, like, the doctors are there (Patient 050).

In this particular case, the choice was between better access to care (the city) and a safe place for grandchildren to grow (the reserve).

Once it was clear that treatment was not going to result in remission, family members discussed the challenges associated with taking their relative home (Patients 005, 031, 209 and 210). In most cases, care was provided by family members and local healthcare staff, until death was imminent and pain control or other symptoms required medical care.

[W]e decided [that I would care for her at home]… If you can control the pain, it’s – it’s okay… So we fixed everything up at home. We had the bed that can go up and down, the bathroom, a little stool beside the bed. We made it into a little hospital… I got to learn how to work those oxygen bottles; they’re only good for 4 h, and a little machine for her to breathe. We – we pulled it through for about 9 months. And then, 1 day she fell down and she said, “I don’t think I’m gonna make it.” She said, “I’m very, very sick.” ‘Cuz she went right down to her frail bones… I wasn’t mad at her; I was mad at myself because this – this cancer thing – it’s terrible for everybody – anybody. And took her to the [regional] hospital and 5 days later, she passed on (Patient 005).

For others still, coming home was not possible, for a variety of reasons, including the lack of appropriate clinical support in the home community. These were complex decisions, particularly when elderly peoples or elders within the community tried to advocate for their own relocation home:

[S]ometime we got her really mad when we told her we couldn’t take her because the doctors said. She said, “I’m not married to the doctor” (Patient 209).

In some cases, going home for a last visit was accepted as a compromise.

[My daughter]… wanted to come home for the last time. She said, “I wanna go home and visit”, you know? And so, what happened was: I went to the casino and won $1500 so we paid our fare… So that’s what she wanted. That was her last wish: to come home and spend a weekend home. That was about 3 weeks before she passed on (Patient 029).

## Discussion

The objective of this paper was to critically explore how the contexts of First Nation peoples’ lives intersect with structural barriers to shape their access to care and expectations of cancer care. We also wanted to highlight policy issues shaping healthcare outcomes. There are several strengths and limitations in this study. A key limitation is that findings reported here are based on interview data. It was beyond the scope of this paper to link interview data to chart reviews, through this may have provided greater clarity on time lines and patients trajectories. This work was however not pursued, as we would have had to secure access to multiple patient charts (including community file, the FP, regional hospitals, Winnipeg-based hospitals). Until integrated electronic medical records are in place, this type of study will have to depend on interviews alone.

Our findings show that logistical fatigue often occurred after treatment was initiated. Patient and family member narratives also reinforced the need to maintain family and community connections, are key determinants of First Nation decision-making with regard to treatment options, location of treatment and overall adherence. Late diagnosis despite repeated consultations results in a need for more invasive treatments, available in Winnipeg only. Some patients chose not to pursue acute cancer care treatment and remain with their loved ones, with the hope to die at home. Others opt for relocation to the city to be closer to care. Still, the majority of participants and family members interviewed for this paper commuted to receive care. All participants reported logistical fatigue, and many suggested that this led to compromise in the pursuit of care. The main reasons cited for making compromises in adherence related to the need to balance family, community and cultural obligations with treatment needs. While some compromises are unavoidable, financial pressures, a lack of coordination in services offered by different jurisdictions, and a lack of attention to the patient’s circumstances in the care plan played a key role. In the context of Manitoba First Nations, a whole system’s approach is required to address cancer care needs.

Although all Manitobans residing in rural and remote areas must also relocate for specialized cancer care treatment [27], the socio-historical and jurisdictional contexts influencing First Nation peoples’ access to healthcare create a unique set of complexities [16, 28–30]. The findings discussed in this paper make visible a particular set of structural barriers to timely diagnosis, financial barriers to pursue care, factors impacting decisions to pursue or interrupt care, and complexities surrounding navigating the cancer care system. These findings link to existing policies that at times attempt to mitigate, but often magnify barriers to care.

Discourses of consumerism in health care [31], increased self-management, concepts of patient empowerment and self-efficacy, and health literacy [32] are increasingly more prevalent in the healthcare literature. Patients and families facing barriers to care are increasingly expected to use self-advocacy in their navigation of the system. We see a number of issues with these discourses and concepts. To begin, being assertive can be difficult given past and often intergenerational experiences of dismissal when seeing healthcare services [11, 13, 33]. For Manitoba First Nations, experiences of dismissal remain very much in the present, with catastrophic results [34, 35].

Patient navigator programs have been shown elsewhere to have a significant positive impact on continuity of care where barriers are related to patient’s attempts at navigating an existing and functional system [25]. Patient navigators will likely have limited success in cases where barriers to care are systemic [36], and in this case, related to federal-provincial wrangling over roles and costs. This is particularly true where patient navigators are employees or contactors of the system creating barriers.

Sadly, our findings are not unique. A recent review of cancer incidence in Indigenous peoples in Australia, New Zealand, Canada and the United States reported that,

The high incidence of certain cancers in indigenous [sic] people fits the pattern of high incidence of disease and infection related to social deprivation in low-income and middle-income countries; our findings highlight the common legacy of colonisation and its resultant political, social, environmental, and economic effects on the health of indigenous people [37].

In Australia, social exclusion (racial inequity, poverty and related disadvantage) was identified as a key barrier to pursuing care [38]. Although health literacy was also mentioned as an important factor [39–41], our data suggest that what may be construed as a health literacy deficit (for example, a belief that cancer is a death sentence) may be an accurate reflection of Indigenous peoples’ reality given structural barriers. Cultural explanations should not overshadow the need for structural change.

## Conclusions and recommendations

Our findings suggest a number of “missed opportunities” where improved access to early diagnosis, better policy and program alignment and increased responsiveness to patients and family circumstances could result in more manageable care trajectories for patients and families, reduced logistical fatigue and possibly better outcomes.

### Enhance access to early diagnostic pathways

Many patients experienced barriers to early diagnosis, despite repeated attempts at seeking care. These barriers are the results of multiple intersecting factors including the lack of availability for screening opportunities in rural, remote and on-reserve communities [21, 27]; poor access to a transportation system to seek screening off-reserve [7]; and limiting scope of practice regulations [often understood as a lack of capacity on-reserve, rather than restrictive regulations, see 42] which constrain what services on-reserve nursing staff can provide. As discussed earlier, health services provided on-reserve are limited to prevention and public health, delivered by community health nurses and paraprofessionals (Community Health Representatives). Some larger remote communities are served by nursing stations, where nurses working on a broader scope of practice provide selected primary care services.

On-reserve facilities do not have the capacity to provide screening and diagnostic services. Access to diagnostic services located off-reserve requires a referral from these nurses or from a visiting FP. Only selected First Nation communities have visiting FPs..FNIHB requires a referral to provide support for medical transportation. These factors result in late diagnoses, much more expensive treatments, human tragedy, premature mortality and avoidable healthcare costs. Those interviewed reported having repeatedly attempted to seek diagnosis, only to be repeatedly dismissed.

A key to improving cancer outcomes for First Nation peoples living on-reserve is to ensure timely access to diagnostic services. This will require a) a partnership between FNIHB and cancer diagnostic services to include screening on-reserve; b) a change in scope of practice for nurses working on-reserve; and c) since not all cancers can be screened for or diagnosed on-reserve, a change in the medical transportation policy to include medical transportation for diagnostic care.

### Cross-jurisdictional case management

Despite governmental commitments made to Jordan’s Principle, cross-jurisdictional case management remains underdeveloped and unsupported by policy for all First Nation peoples including children [34]. Increasingly stringent accountability frameworks for government programs [13], coupled with dwindling budgets, have created inflexibilities and diminished opportunities for patient-centric responses. This trend is a disservice to First Nations, and undermines responsiveness and, as a result, adherence. Recent attention to the Jordan Principle will hopefully result in progress towards jurisdictional policy coordination, a requirement to improving First Nation peoples access to cancer care.

### Address the broader context of peoples’ lives and healthcare needs

Although the providers we interviewed expressed concerns for their First Nation patients, many also expressed powerlessness in making services more readily accessible or responsive. Participants reported that the AMC patient navigator and the Winnipeg Regional Health Authority social worker programs have been invaluable in helping them navigate access to health and social services, housing, income assistance, and other services once in the city. Patient navigation programs have been promoted in Canada [6] and elsewhere [25, 43], and the need for this type of patient advocacy has been articulated for decades [44]. Although this is a step in the right direction, all mentioned that their case load is too large to be able to provide the level of case management required. As a result, other care providers must take on the role of advocating for their patients, and while some do, it is clear from the interviews discussed in this paper that providers’ level of engagement in advocacy varies, depends largely on the understanding and goodwill of the provider, and is vulnerable to logistical fatigue as well as competing demands on time. Although resolving cross-jurisdictional issues at the policy level, as discussed above, will improve access and might diminish pressures on existing patient navigation programs, this will take some time. In the short term, investment in patient navigation programs is required to ensure manageable caseloads and adequate support.

## Abbreviations

CCMB: Cancer care Manitoba
FNIHB: First Nations and Inuit Health Branch of Health Canada
FPs: Family Physicians (FPs)
NIHB: Non-insured health benefit program
WRHA: Winnipeg Regional Health Authority

## Footnotes

1 The prescription drug formulary for the NIHB program is defined nationally, and implemented for all First Nations no matter where they live. In contrast, all Canadian provinces define their own prescription drug formulary for coverage. Important discrepancies between provinces have been noted especially for cancer drug coverage [45]. For example, Manitoba Health currently provides free access to cancer drugs for all Manitoba residents, unless they are First Nations. First Nations are expected to get their cancer drug coverage from the federal–funded NIHB program. The NIHB formulary however includes only a few cancer drug options, leaving Manitoba First Nations with few free treatment options when compared to other Manitobans.
